# Captivity Influences Gut Microbiota in Crocodile Lizards (*Shinisaurus crocodilurus*)

**DOI:** 10.3389/fmicb.2020.00550

**Published:** 2020-04-23

**Authors:** Guo-Shuai Tang, Xi-Xi Liang, Meng-Yuan Yang, Ting-Ting Wang, Jin-Ping Chen, Wei-Guo Du, Huan Li, Bao-Jun Sun

**Affiliations:** ^1^Key Laboratory of Animal Ecology and Conservation Biology, Institute of Zoology, Chinese Academy of Sciences, Beijing, China; ^2^University of Chinese Academy of Sciences, Beijing, China; ^3^State Key Laboratory of Cardiovascular Disease, Fuwai Hospital, National Center of Cardiovascular Disease, Chinese Academy of Medical Sciences and Peking Union Medical College, Beijing, China; ^4^Guangdong Key Laboratory of Animal Conservation and Resource Utilization, Guangdong Public Laboratory of Wild Animal Conservation and Utilization, Guangdong Institute of Applied Biological Resources, Guangzhou, China; ^5^Institute of Occupational Health and Environmental Health, School of Public Health, Lanzhou University, Lanzhou, China

**Keywords:** *Shinisaurus crocodilurus*, gut microbiota, age, captive population, wild population, wild animal conservation

## Abstract

Captivity is an important measure for conservation of an endangered species, and it is becoming a hot topic in conservation biology, which integrates gut microbiota and endangered species management in captivity. As an ancient reptile, the crocodile lizard (*Shinisaurus crocodilurus*) is facing extreme danger of extinction, resulting in great significance to species conservation in the reserve. Thus, it is critical to understand the differences in gut microbiota composition between captive and wild populations, as it could provide fundamental information for conservative management of crocodile lizards. Here, fecal samples of crocodile lizards were collected from two wild and one captive populations with different ages (i.e., juveniles and adults) and were analyzed for microbiota composition by 16S ribosomal RNA (rRNA) gene amplicon sequencing. This study showed that the lizard gut microbiota was mainly composed of Firmicutes and Proteobacteria. The gut microbiota composition of crocodile lizard did not differ between juveniles and adults, as well as between two wild populations. Interestingly, captivity increased community richness and influenced community structures of gut microbiota in crocodile lizards, compared with wild congeners. This was indicated by higher abundances of the genera *Epulopiscium* and *Glutamicibacter*. These increases might be induced by complex integration of simple food resources or human contact in captivity. The gut microbiota functions of crocodile lizards are primarily enriched in metabolism, environmental information processing, genetic information processing, and cellular processes based on the Kyoto Encyclopedia of Genes and Genomes (KEGG) database. This study provides fundamental information about the gut microbiota of crocodile lizards in wild and captive populations. In the future, exploring the relationship among diet, gut microbiota, and host health is necessary for providing animal conservation strategies.

## Introduction

Bringing animals into captivity and maintaining breeding populations in natural reserves is an important measure undertaken to protect the declining biodiversity of endangered species (Redford and Mcaloose, 2012). For example, the crested ibis *Nipponia nippon* was once thought extinct before seven individuals were rediscovered in 1981. After captive breeding, the individual number increased to more than 200, including 130 in captivity by 2000 (Xi et al., 2002). Meta-analysis of marine reserves indicates that there are 3.7 times more fish populations inside the reserves than outside (Mosquera et al., 2000). Furthermore, the panda reserve system in China provides one of the highest biodiversity among temperate regions worldwide (Mackinnon, 2008; Li and Pimm, 2016). Given the control of fundamental information of species and scientific management by the scientific community, capacity and breeding populations in natural reserves can effectively manage and conserve endangered species and their biodiversity (Ebenhard, 1995).

The crocodile lizard (*Shinisaurus crocodilurus* Ahl, 1930) is a monotypic species in the genus *Shinisaurus* and monotypic family Shinisauridae, which is remnant of an ancient lineage from the Pleistocene with around 200 million years of history (Zhao et al., 1999). Because of their narrow distribution, small population, being heavily hunted, and environmental changes, it is listed as a class I protected species in China. The International Union for Conservation of Nature (IUCN) Red List of Threatened Species also list *S. crocodilurus* as an endangered species (Nguyen et al., 2014). What is more, it was listed as appendix I species (CITES I) by the Convention on International Trade in Endangered Species of Wild Fauna and Flora (Schingen et al., 2016). Consequently, the current captive reserve is one of the most effective protection strategies for crocodile lizards (Huang et al., 2008; Van Schingen et al., 2015).

During capacity, the fundamental information of crocodile lizard, including its genetic classification (Huang et al., 2014, 2015), morphological structure (Conrad, 2006), habit distribution (Wu et al., 2012; Huang et al., 2014), and artificial breeding (Wang et al., 2008; Yu et al., 2009), has been revealed gradually. These studies have provided great information about crocodile lizards for captive breeding and conservation. However, like other captive species, some serious challenges are posed by the crocodile lizards during capacity in the nature reserves (Snyder et al., 1996). For example, the captive population has been plagued by various unknown diseases, nutritional deficiency, and low reproductive rates (Jiang et al., 2017).

In recent years, with rapid development of high-throughput sequencing, an increasing number of studies interpreted the health and nutritional utilization of animals by integrating the relationships between bacteria in gastrointestinal tracts and the animals themselves (Mcfall-Ngai et al., 2013). For instance, the host’s genotype (Kovacs et al., 2011; Goodrich et al., 2014), age (Elena et al., 2010; Yatsunenko et al., 2012; Jian et al., 2015), health (Dethlefsen et al., 2007), dietary composition (Castillo and Martín, 2007; David et al., 2013; Zhang et al., 2014), and even social interaction (Lombardo, 2008) can determine the gut microbial composition of animals. Meanwhile, the composition of the gut microbiome in an animal can affect its health status (Clemente et al., 2012; Martín et al., 2014), metabolism (Ramakrishna, 2013), immunity (Thaiss et al., 2016), and coevolution of the host (Ley et al., 2008; Moeller et al., 2016). Thus, promoting the conservation of endangered species by studying gut microbiota has been receiving increasing attention and has become a hot topic of conservation biology (e.g., Zhu et al., 2011; Wu et al., 2017). With limited studies conducted on the lizard gut microbiota, factors such as diet (Hong et al., 2011; Jiang et al., 2017), gender (Martin et al., 2010), adaptation (Ren et al., 2016), captive breeding (Kohl and Dearing, 2014; Kohl et al., 2017), and even climate change (Bestion et al., 2017) have been demonstrated to affect the gut microbiota.

As the gut microbiota is tightly associated with host health and physiology, it is critical to understand the differences in gut microbiota composition in crocodile lizards between captive and wild populations during the processes of conservation. It remains unknown whether captivity can influence gut microbiota and thus influence animal health. This comparison is not only important to understanding the gut microbiota variation but also critical to providing conservation insight into endangered species conservation in captivity. In addition, captive conservation should be related to multiple stages of life history, including adults and juveniles. In particular, juveniles are more vulnerable to challenges currently confronting captive crocodile lizards (i.e., diseases and nutritional deficiency). It has been known that age-dependent gut microbiota is important to digestibility and consequently to conservation efforts (Redford and Mcaloose, 2012; Jian et al., 2015). Therefore, in order to explore whether the gut microbiota composition of the crocodile lizard varies along ages and captive environment, it is necessary to analyze its composition of gut microbiota between captive and wild environments, as well as between juveniles and adults.

Here, fecal samples of crocodile lizards with different ages were collected from captive and wild populations. We aimed to determine variations in gut microbiota of crocodile lizards between wild and captive environments, as well as between juveniles and adults, using 16S ribosomal RNA (rRNA) gene sequencing of gut microbiota. In addition to promoting the conservation of this endangered species, it provides further insight into the ecological and evolutionary relationship between reptiles and their gut microbiota.

## Materials and Methods

### Sample Collection

**FIGURE 1:**
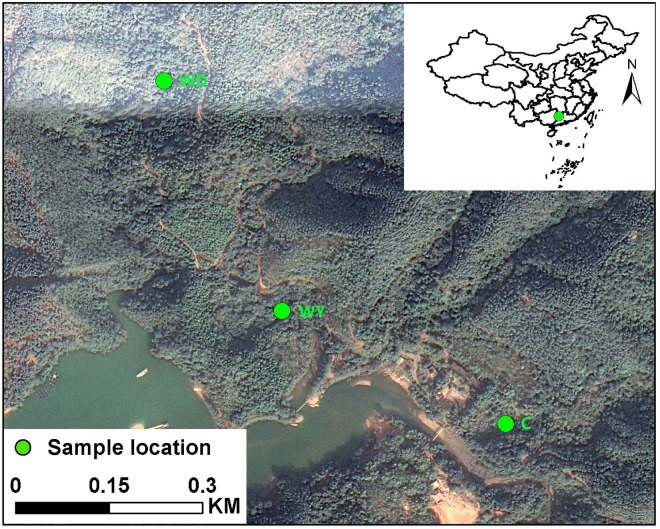
Location of Yusan stream population (WY), Dachai stream population (WD), and captive population (C) in the Crocodile Lizard National Nature Reserve.

Fecal samples of 31 crocodile lizards were collected from Guangxi Daguishan *S. crocodilurus* National Nature Reserve, Guangxi Province, China. These 31 crocodile lizards were from the Yusan stream (*N* = 10), Dachai stream (*N* = 10), and captive populations (*N* = 11), respectively (Figure 1). For each population, fecal samples from both juveniles and adults were collected according to body sizes, respectively. The snout-vent lengths (SVLs) were 161.46 ± 1.98 (151–173) and 106.70 ± 1.69 (98–117) mm, and body masses (BMs) were 90.45 ± 3.89 (63.5–112.7) and 29.89 ± 1.41 (20.7–36.9) g for adults and juveniles, respectively. According to the location and age of the crocodile lizards, the fecal samples were from one of six groups: adults in the wild population of Yusan stream (WY1, *N* = 4), juveniles in the wild population of Yusan stream (WY2, *N* = 6), adults in the wild population of Dachai stream (WD1, *N* = 6), juveniles in the wild population of Dachai stream (WD2, *N* = 4), adults in the captive population (C1, *N* = 6), and juveniles in the captive population (C2, *N* = 5) (see details in Supplementary Table S1). The Yusan and Dachai streams are two independent wild ravine streams in the Crocodile Lizard National Nature Reserve (Figure 1). It is plausible that the crocodile lizards of Yusan and Dachai streams are independent from each other without population communication because of the limited dispersal ability and small home range of crocodile lizards and the isolation of the two streams. During collection, the diet type of two wild populations was randomly investigated. All fecal samples were collected directly without touching anything (Wang et al., 2016a). After collection, the fecal samples were transported back to the laboratory in Beijing with sterile containers. The fecal samples were stored in a −80°C refrigerator before DNA extraction.

### Extracting DNA, PCR Amplification, and Sequencing

All DNA extraction and sequencing were conducted by Novogene Corporation (Beijing, China) with established protocols. In brief, cetyltrimethyl ammonium bromide (CTAB)/sodium dodecyl sulfate (SDS) method was employed for total DNA extraction from the lizard fecal samples. Then, 1% agarose gels was used for concentration and purification of DNA. After, DNA were diluted to 1 ng/μL with bacteria-free water before bacteria 16S rRNA amplification. Barcodes of 341F (5′-CCTAYGGGRBGCASCAG-3′) and 806R (5′-GGACTACNN GGGTATCTAAT-3′) were the primers for amplification of the V3–V4 region of the bacteria 16S rRNA gene. Thermal cycling conditions of the PCR assay were as follows: 1 min initial denaturation at 98°C, 30 cycles of 10 s denaturation at 98°C, 30 s annealing at 50°C, finally 30 s elongation at 72°C, and a final extension at 72°C for 5 min. A 30-μL reaction system was used for PCR products, which contained 10 ng template DNA, forward and reverse primers (0.2 μM), and 15 μL Phusion^®^ High-Fidelity PCR Master Mix (New England Biolabs, United Kingdom). The GeneJET^TM^ Gel Extraction Kit (Thermo Scientific, United States) was used for sufficient mixture and purification of the obtained amplification products. Then, with Ion Plus Fragment Library Kit (Thermo Scientific, United States), the sequencing libraries were established according to published protocols of the kit. After establishment, the libraries were measured on the Qubit^®^ 2.0 Fluorometer (Thermo Scientific, United States). After, an Ion S5TM XL platform was used to sequence the library, with 400/600 bp single-end reads generated. Obtained raw sequences were submitted to the National Center for Biotechnology Information (NCBI) Bioproject database (accession number PRJNA594801) (See details in Supplementary Data Sheet 1).

### Data Analysis

#### Clean Raw Data

The tags of raw sequences were filtered using Cutadapt (V1.9.1^1^) (Martin, 2011). All sequences were compared on UCHIME algorithm to find out chimera sequences (UCHIME Algorithm^2^) (Edgar et al., 2011), with Silva database as reference (Silva database^3^) (Quast et al., 2013). After filtering out all low-quality and chimera sequences, the remaining clean reads were obtained.

#### OTU Production

Sequences were assigned with similarity no less than 97% (i.e., ≥97%) to the same operational taxonomic units (OTUs) using Uparse (Uparse v7.0.1001^4^) (Edgar, 2013). For each OTU, we searched the Silva Database^5^ to annotate screened representative sequence with threshold 0.8 using RDP Classifier 2.2 (Wang et al., 2007; Quast et al., 2013).

#### Data Normalization

In order to compare different samples, the number of the samples with the lowest counts was used to normalize the OTU abundance information. The rarefaction curves of observed species were calculated to assess the sufficiency of current depth of sequencing, in yielding a stable estimate of the species richness. Whether the bacterial diversity in the 31 fecal samples represents the overall bacterial diversity in the gastrointestinal tract of the crocodile lizard was determined with a species accumulation box plot.

#### Alpha and Beta Diversity Estimation

The observed-species index and Simpson index was calculated with QIIME V1.7.0 to estimate alpha diversity for each fecal sample of crocodile lizard (Caporaso et al., 2010), which were indicators in community richness and community evenness identifications, respectively. Then, the Mann–Whitney *U*-test was performed to detect differences in alpha diversity indices between two independent groups.

For the beta diversity metrics, principal component analysis (PCA) and analysis of similarities (ANOSIM) were conducted to determine the communities and structure of the gut microbiota among groups. PCA, which is based on the OTU level, can intuitively present the differences among groups on a two-dimensional graph. Notably, ANOSIM based on the Bray–Curtis distances, considered both flora types and the relative abundance of microbes.

The differential abundances were compared at family and genus levels of bacteria among groups using LEfSe analysis to identify microbes accounting for the effect of captivity or age. Thereafter, a set of pairwise tests was used to investigate biological consistency among subgroups. The linear discriminatory analysis (LDA) was also performed to evaluate the effect size of each selected classification. In this study, only bacterial taxa with a log LDA score more than 4 (more than four orders of magnitude) were used (Segata et al., 2011).

#### Functional Classification

Functional prediction of the sequences among groups was conducted for classification. In brief, PICRUSt was utilized to search the protein sequences of the predicted genes in the Kyoto Encyclopedia of Genes and Genomes (KEGG) database with *E* value < 1E-5. These genes were assigned to KEGG pathways (Langille et al., 2013). Then, relative abundance in each group was counted. The unique and shared genes between populations were also plotted by Venn diagram. A heatmap was used to show genes with high expression.

## Results

### Food Composition of Wild and Captive Populations

The primary food types of Yusan and Dachai stream populations were similar, mainly consisting of earthworm, centipede, and larvae of lepidopteran, which comprised around 70% of the food availability. In contrast, the earthworm is the only food type for captive crocodile lizards during breeding (Table 1).

**TABLE 1:** Primary food types of wild and captive crocodile lizards.

Proportion of composition	Captive lizards	Wild lizards
Most	Earthworm	Earthworm
Secondary		Centipede
Tertiary		Larva of Lepidoptera
Other		Larva of other insects

*Food types are shown at category levels, and rough proportion of food compositions is shown in sequence.*

### General Analyses of the Gut Microbial Community Structure

The bacterial composition of 31 crocodile lizard fecal samples was analyzed (Supplementary Table S1). The average effective sequences of 31 samples were 52,342 (Supplementary Figure S1). The estimates of species richness were stable and unbiased according to the rarefaction curves (Supplementary Figure S2). The species accumulation boxplot indicated that the sample size was sufficient and greatly saturated the bacterial diversity found under this condition (Supplementary Figure S3).

**FIGURE 2:**
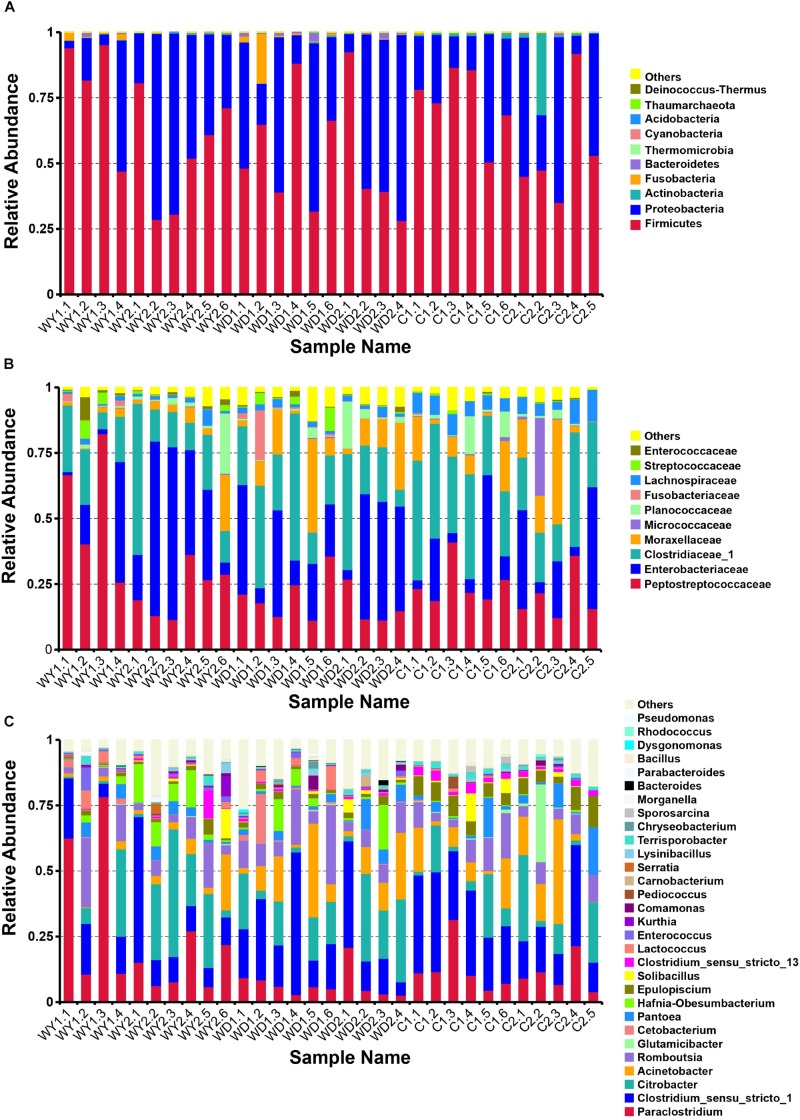
Composition of the gut microbiota of each sample at the **(A)** phylum, **(B)** family, and **(C)** genus levels. Different colors in the figures indicate the different groups, and details are shown on the right sides of each figure, respectively. Details of sample names are shown in Supplementary Table S1. In each panel, “Others” represented the sum of the relative abundances of all other phylum **(A)**, families **(B)**, and genus **(C)** except the items listed in the figure.

The total sequences of crocodile lizards were classified into five major phyla (Figure 2A), Firmicutes, with the relative abundance of 61.2%, holding the overwhelming predominance. Proteobacteria (35.8%), Actinobacteria (1.4%), Fusobacteria (1.0%), and Bacteroidetes (0.5%) were the other four major phyla. Totally, these five most dominant phyla contributed more than 99% abundance across all the samples. At the family level, the top 10 families are listed (Figure 2B). The most abundant taxa were *Peptostreptococcaceae* (25.5%), *Clostridiaceae_1* (25.3%), *Enterobacteriaceae* (25.0%), and *Moraxellaceae* (9.3%). In addition, the top 30 genera are also listed (Figure 2C). The gut microbiota of all these crocodile lizards was dominated by *Clostridium sensu_stricto 1* (21.0%), *Citrobacter* (14.8%), *Paraclostridium* (14.3%), *Acinetobacter* (9.3%), and *Romboutsia* (9.1%).

### Comparison of Gut Microbial Community Structure Between Age or Populations

**FIGURE 3:**
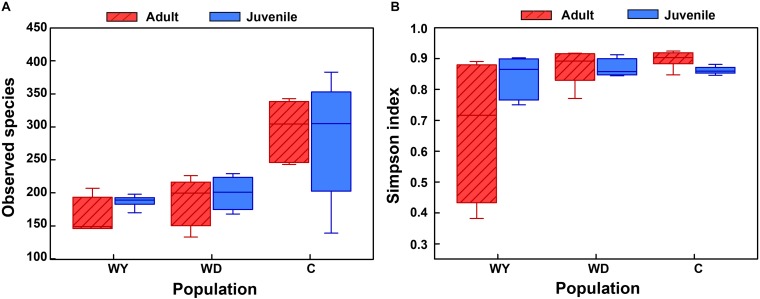
The alpha diversity of the gut microbial composition, shown by observed species index **(A)** and Simpson index **(B)** among populations. WY indicates wild population of Yusan stream, WD indicates wild population of Dachai stream, and C indicates captive population. Data are expressed as mean ± SEM.

**FIGURE 4:**
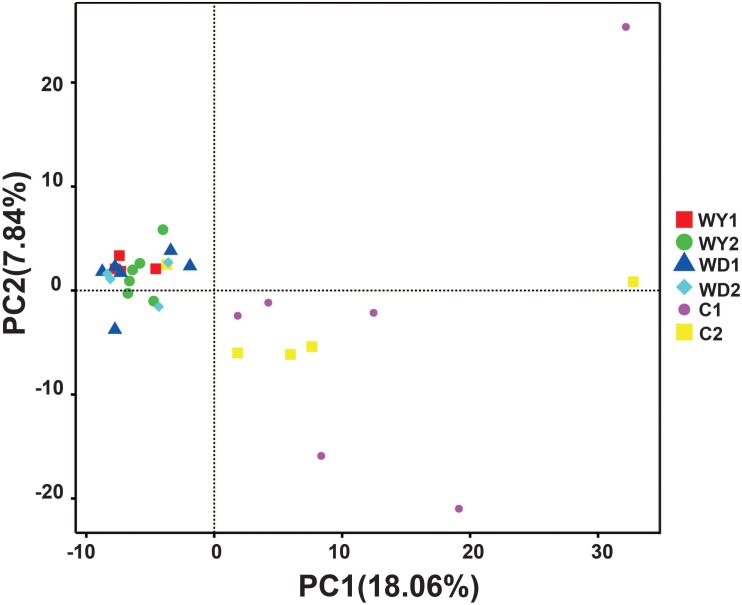
The beta diversity of the gut microbiota composition of two wild populations and captive population. Principal component analysis (PCA) was performed. The variation explanation is indicated on each axis, respectively.

**FIGURE 5:**
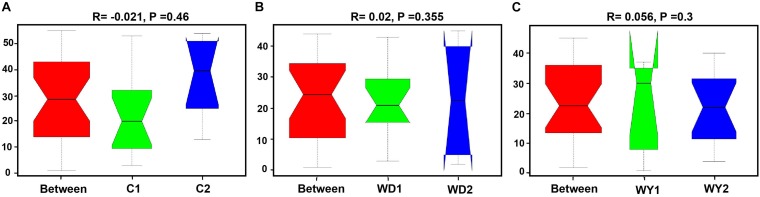
Analysis of similarity between adults and juveniles of **(A)** captive, **(B)** WD, and **(C)** WY populations. **(A)** C1 and C2 indicate the adults and juveniles of captive population, **(B)** WD1 and WD2 indicate the adults and juveniles of wild Dachai population, and WY1 and WY2 indicate the adults and juveniles of wild Yusan population, respectively. Data are expressed as mean ± SEM. Statistical significance is defined as α < 0.05.

**FIGURE 6:**
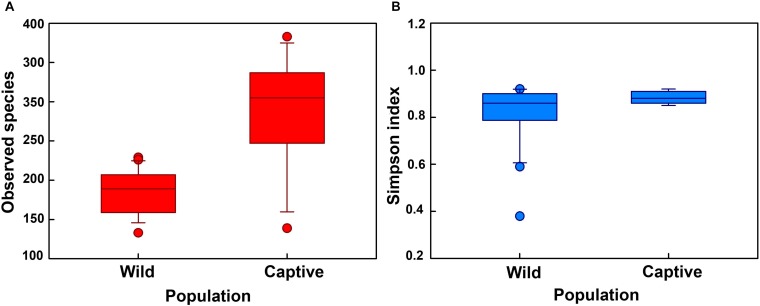
The alpha diversity of the gut microbial composition, shown by observed **(A)** species index and **(B)** Simpson index between wild and captive populations. “Wild” indicates combination of wild populations from Yusan and Dachai streams, and “Captive” indicates captive population. Data are expressed as mean ± SEM.

First, the gut microbial diversity was compared between adult and juvenile crocodile lizards within each population, respectively. No significant difference between the adult and juvenile individuals was identified in terms of community richness (Figure 3A), community evenness (Figure 3B), or community composition (Figure 4) (all *P* > 0.05). ANOSIM also indicated similarity between adult and juvenile individuals in each population (all *P* > 0.05) (Figure 5). Integrated in the results of alpha and beta diversity analyses, the gut microbiota of adults and juveniles within each population were highly similar, respectively. Therefore, adults and juveniles from each population were combined as available individual candidates to compare the variation in gut microbiota at the population level. Accordingly, data analysis was reconducted and recalculated to elucidate the difference in alpha diversity and beta diversity using population as main factor. The results indicated that the community richness of the captive population was clearly higher than wild populations of Yusan stream (*Z* = −3.170, *P* < 0.05) and Dachai stream (*Z* = −3.239, *P* < 0.05), but no significant difference was detected between two wild populations (*Z* = −1.362, *P* = 0.173) (Figure 3A). After combination of two wild populations, a significant difference was detected between wild and captive populations in community richness (*Z* = 2.412, *P* = 0.016) (Figure 6A). However, no significant difference was detected between wild and captive populations in the community evenness (*Z* = 0.949, *P* = 0.343) (Figure 6B). With regard to beta diversity, the results of the PCA plot and ANOSIM showed significant differences between the captive population and two wild populations, respectively (C-WY, *R* = 0.2935, *P* < 0.05; C-WD, *R* = 0.2929, *P* < 0.05), with similarity between two wild populations (*R* = 0.08122, *P* = 0.128) (Figure 4).

A comparison of the gut microbiota between the wild and the captive populations showed in wild populations, the composition of the gut microbiota mainly includes Firmicutes (60.1%), Proteobacteria (37.6%), Fusobacteria (1.4%), Bacteroidetes (0.7%), and Actinobacteria (0.2%) at the phyla level; Peptostreptococcaceae (28.3%), Enterobacteriaceae (27.9%), Clostridiaceae_1 (22.4%), and Moraxellaceae (8.0%) at the family level; and *Clostridium_sensu_stricto_1* (18.7%), *Paraclostridium* (17.2%), *Citrobacter* (16.4%), *Romboutsia* (9.7%), and *Acinetobacter* (8.0%)at the genus level. In the captive population, the composition of the gut microbiota mainly include Firmicutes (64.2%), Proteobacteria (31.6%), Actinobacteria (3.9%), and Bacteroidetes (0.2%) at the phyla level; Clostridiaceae_1 (29.3%), Peptostreptococcaceae (22.7%), Enterobacteriaceae (19.0%), and Moraxellaceae (11.6) at the family level; and *Clostridium_sensu_stricto_1* (24.0%), *Paraclostridium* (11.7%), *Citrobacter* (11.7%), *Acinetobacter* (11.6%), and *Romboutsia* (7.9%) at the genus level.

### LEfSe Analysis of the Differential Microbes Between Captive and Wild Populations

**FIGURE 7:**
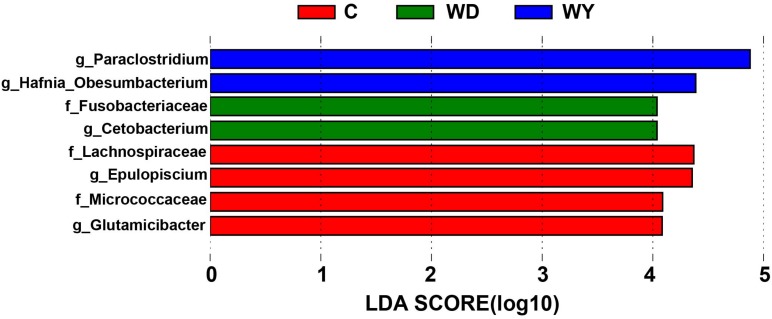
Differences in bacterial taxa among populations determined by linear discriminative analysis of effect size (LEfSe). The highlighted taxa were significantly enriched in the group that corresponds to each color. Linear discriminatory analysis (LDA) scores can be interpreted as the degree of difference in relative abundance. The letters “g” and “f” indicate genus and family, respectively.

The LEfSe analysis indicated that five genera and three families were enriched differently in captive and wild populations. In contrast to wild populations, the gut microbiota of captive crocodile lizards showed significantly higher abundances in genera *Epulopiscium* and *Glutamicibacter*, and in families *Lachnospiraceae* and *Micrococcaceae* (Figure 7).

### Functional Predictions of Gut Microbiota Between Captive and Wild Populations

**FIGURE 8:**
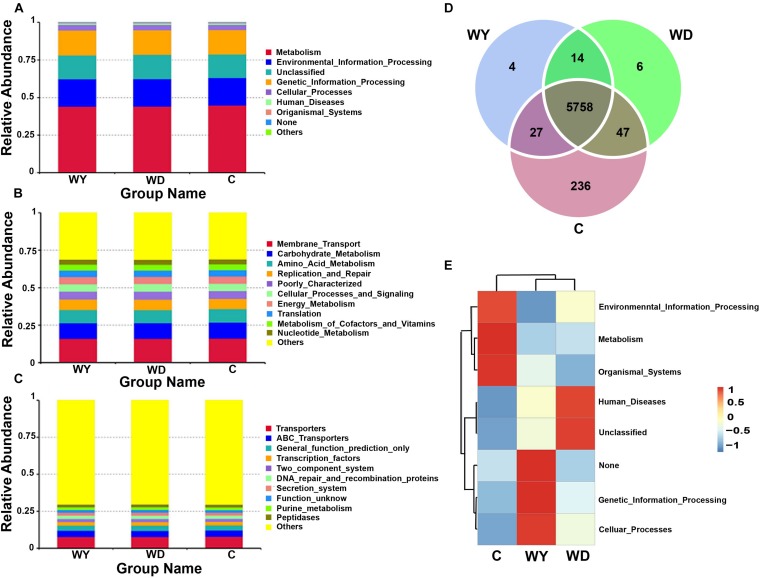
Functional classifications of 16s RNA in microbiota at **(A)** top level, **(B)** second level, and **(C)** third levels of relative abundance, and **(D)** Venn and **(E)** clusters analysis of functions between captive and wild populations. C indicates captive population, WD indicates wild Dachai population, and WY indicates wild Yusan population, respectively.

16S RNA of gut microbiota from 31 fecal samples were predicted into three levels in functional categories. At the top level, metabolism, environmental information processing, genetic information processing, and cellular processes were four primary categories (Figure 8A); at the second level, membrane transport, carbohydrate metabolism, amino acid metabolism, replication and repair, cellular processes and signaling, energy metabolism, translation, metabolism of cofactors and vitamins, and nucleotide metabolism were the primary functions (Figure 8B); while at the third level, transporters, ATP-binding cassette (ABC) transporters, and transcription factors were the primary functions (Figure 8C).

Venn diagram of shared genes indicated that most of the knockouts (KOs) were common in captive and two wild populations, while 236 KOs were exclusive to the captive population (Figure 8D). Heatmap of the cluster indicated that at the top level, the KOs of captive population were enriched in environmental information processing, metabolism, and organismal systems (Figure 8E). However, no significant differences among groups were found after statistical analysis (minimum *P* = 0.270).

## Discussion

Firmicutes and Proteobacteria were two major gut microbiotas in crocodile lizard, while Actinobacteria, Fusobacteria, and Bacteroidetes were minor gut microbiotas. Like other studies, Firmicutes and Proteobacteria are two of the most important types of gut microbiota in numerous vertebrate species (Xenoulis et al., 2010; Waite et al., 2012; Wang et al., 2016b). Phylum Firmicutes (33.2–73%) have been documented as the dominant gut microbiota in lizards, while Proteobacteria (5.7–62.3%) and Bacteroidetes (6.2–45.7%) were varied among host species (Nelson et al., 2010; Hong et al., 2011; Ren et al., 2016; Kohl et al., 2017). In addition, the gut microbiota is similar to lizards in other reptile categories (Costello et al., 2010; Colston et al., 2015; Yuan et al., 2015). Interestingly, previous study on gut microbiota of crocodile lizards in wild and captive populations reported that Proteobacteria and Bacteroidetes were primary, while the proportion of Firmicutes is lower than was detected in this study (Jiang et al., 2017). The potential reason for the discrepancy between the two studies could be the sampling methods used. Jiang et al. (2017) used cloacal swabs for sampling, whereas fecal sampling was utilized in the present study. It has been demonstrated that the fecal communities were largely similar to hindgut microbial communities in lizards, thus becoming an acceptable indicator in the gut region for microbial diversity (Kohl et al., 2017). The communities of cloacal swabs have clear microbial community characteristics, especially in terms of community members, from the communities of large intestine (Colston et al., 2015). Therefore, different sampling methods may lead to variation in gut microbiota. Given that, based on the previous study (Jiang et al., 2017), we provided further understanding of gut microbiota in the crocodile lizards.

The effects of age in crocodile lizards on the gut microbiota were revealed to be trivial, either in the captive or in the wild environments (Figure 5). This may be largely due to the fact that adult and juvenile crocodile lizards of each population live in the same environmental conditions, and food intake is identical accordingly. In the wild, earthworm is a conservatively primary food resource for crocodile lizards (Ning, 2007). In contrast, comparing with the microbial communities of adults, juveniles are usually different, as they are greatly dependent on environments and resources. The difference in environmental dependence of gut microbiota along ontogeny implies more self-governing in adults after maturation (Trosvik et al., 2010; Burns et al., 2016). The crocodile lizard survives independently after birth, so the gut microbiota of juvenile individuals may be similar to adults. In addition to the neutral effect of age on gut microbiota, the gut microbiota did not differ in two wild populations. This similarity is accompanied with homologous food composition between two wild populations (Table 1); however, they are isolated. In the field, diet may be one of the most important factors affecting the composition of the gut microbiota of wild animals (Wu et al., 2011; David et al., 2013). In the future, it would be interesting to reveal the effect of ontogeny on gut microbiota variation in crocodile lizard, which could provide more insight.

Most interestingly, the captive population was found to modify the community structure and had higher community richness than the two wild populations (Figure 6A). This study found a contrasting pattern to those studies that demonstrated lower microbial diversity in animals under captivity (Kohl and Dearing, 2014; Kohl et al., 2014) or those that found similar gut microbiota between wild and captive lizards (Wang et al., 2016c; Kohl et al., 2017). Although a number of studies have demonstrated that the gut microbiota of animals in captive are different from congeners in the wild (Villers et al., 2010; Xenoulis et al., 2010; Wienemann et al., 2011; Nelson et al., 2013), captive population is seldom detected to have higher community richness than wild populations. It might be led by different food composition between captive and wild populations (Table 1). The food types are more diverse in wild environments than in captive environments for crocodile lizards. Diet is one of most important factors that affect the assembly of gut microbiota (Muegge et al., 2011; Carmody et al., 2015; Pérez-Cobas et al., 2015). However, most of the studies indicated positive relationships between food and gut microbiota diversities (e.g., Laparra and Sanz, 2010; Li et al., 2016). In contrast, captive crocodile lizard has an opposite pattern. The underlying mechanisms are still largely unknown. Future studies with food type manipulations would be helpful to reveal their relationships.

In captivity, constant cohabitation, social interaction, and interaction with human keepers provide increased opportunities for transmission of microbiota from host-associated sources, which are capable of colonizing the animals. This, in turn, may contribute to the increased community richness in the gut microbiota of the captive animals (Nelson et al., 2013). In this study, the captive lizards had higher abundances of families *Lachnospiraceae* and *Micrococcaceae* and genera *Epulopiscium* and *Glutamicibacter* than wild lizards. Among them, *Lachnospiraceae* (phylum Firmicutes, order Clostridiales) is typically abundant in the digestive tracts of humans, ruminants, and many other mammals (e.g., Gosalbes et al., 2011; Sandra et al., 2013). *Lachnospiraceae* has been demonstrated to be related with the production of butyrate, which is necessary to sustain the health of colonic epithelial tissue (Duncan et al., 2002). The captive population of crocodile lizard has more opportunities in contacting with humans, by frequent feeding, cleaning of the breeding pond, examination of diseases, etc., which may result in colonization of the *Lachnospiraceae* bacteria from human. However, whether the increase in *Lachnospiraceae* in abundance has a positive impact on the captive population is still unclear, even though some functional categories for genes of gut microbiota were found in this study in wild and captive populations. More exclusive KOs were found in captive population (Figure 8D), but no significant difference in functions was found. In the future, whole genome sequencing of gut microbiota in crocodile lizards may be helpful at revealing the functions underlying gut microbiota difference between captive and wild populations.

These captivity-related changes in gut microbial communities may have implications for the health of the captive animal and thus determining the success of species conservation (Redford and Mcaloose, 2012). Thus, understanding the effect of the captivity on the composition of gut microbiota is important to provide breeding environments for the health management of the endangered species. This is important, as the composition of the gut microbiome of animals could have long-term effect on their health status and immunity (e.g., Clemente et al., 2012; Martín et al., 2014; Thaiss et al., 2016). It was speculated that the increase in the abundance of these specific bacteria in the captive population may be one of the reasons that affect the survival status of the crocodile lizards. However, the actual relationship in *Lachnospiraceae* by contacting between lizards and humans, and potential function of *Lachnospiraceae* on lizards’ conditions have not been determined to date. Prospectively, the necessity to take actions is recommended in order to minimize the direct contact between human managers and crocodile lizards, including wearing gloves and protection suits during operation on lizards, or sterilizing the equipment used for lizards breeding before operations. Future studies should be centered on the functional interaction between gut microbiota and animals to reveal the functional significance of different richness, as well as the effects of human contact.

## Conclusion

This study revealed the similarity of gut microbiota between adult and juvenile crocodile lizards, both in the captive and wild environments as well as between two wild populations. Interestingly, a significant effect of captivity was found on the composition of gut microbiota of the crocodile lizard, mainly reflected in the increase in community richness and community structure change. After comparison, it was speculated that the gut microbiota variation in captive population might be from human contact. Although the functions are unclear, it was recommended that minimal direct contact was crucial for the health of wild animals between crocodile lizards and human managers in captive environment.

## Data Availability Statement

The datasets generated for this study can be found in the NCBI Bioproject database (PRJNA594801).

## Ethics Statement

The animal study was reviewed and approved by the Animal Ethics Committee at the Institute of Zoology, Chinese Academy of Sciences (IOZ14001).

## Author Contributions

B-JS, G-ST, and W-GD conceived the ideas and designed methodology. G-ST, X-XL, and T-TW collected the data. HL, G-ST, M-YY, and B-JS analyzed the data and led the writing of the manuscript. All authors contributed critically to the drafts and gave final approval for publication.

## Conflict of Interest

The authors declare that the research was conducted in the absence of any commercial or financial relationships that could be construed as a potential conflict of interest.

## Footnotes

1 http://cutadapt.readthedocs.io/en/stable/

2 http://www.drive5.com/usearch/manual/uchime_algo.html

3 https://www.arb-silva.de/

4 http://drive5.com/uparse/

5 https://www.arb-silva.de/
