# Supplementary material for: Captivity Influences Gut Microbiota in Crocodile Lizards (Shinisaurus crocodilurus)
Source: Front Microbiol. 2020 Apr 23;11:550. doi: 10.3389/fmicb.2020.00550 (PMC7190797; doi:10.3389/fmicb.2020.00550)

Table S1. Sample ID in this study. WY means wild population of Yusan stream, WD means wild population of Dachai stream, and C means captive population.

Group	Sample	Age	Source
WY1	WY1.1	Adult	Wild, YuSan stream
	WY1.2	Adult	Wild, YuSan stream
	WY1.3	Adult	Wild, YuSan stream
	WY1.4	Adult	Wild, YuSan stream
WY2	WY2.1	Juvenile	Wild, YuSan stream
	WY2.2	Juvenile	Wild, YuSan stream
	WY2.3	Juvenile	Wild, YuSan stream
	WY2.4	Juvenile	Wild, YuSan stream
	WY2.5	Juvenile	Wild, YuSan stream
	WY2.6	Juvenile	Wild, YuSan stream
WD1	WD1.1	Adult	Wild, DaChai stream
	WD1.2	Adult	Wild, DaChai stream
	WD1.3	Adult	Wild, DaChai stream
	WD1.4	Adult	Wild, DaChai stream
	WD1.5	Adult	Wild, DaChai stream
	WD1.6	Adult	Wild, DaChai stream
WD2	WD2.1	Juvenile	Wild, DaChai stream
	WD2.2	Juvenile	Wild, DaChai stream
	WD2.3	Juvenile	Wild, DaChai stream
	WD2.4	Juvenile	Wild, DaChai stream
C1	C1.1	Adult	Captive
	C1.2	Adult	Captive
	C1.3	Adult	Captive
	C1.4	Adult	Captive
	C1.5	Adult	Captive
	C1.6	Adult	Captive
C2	C2.1	Juvenile	Captive
	C2.2	Juvenile	Captive
	C2.3	Juvenile	Captive
	C2.4	Juvenile	Captive
	C2.5	Juvenile	Captive

Fig. S1 operational taxonomic units (OTUs) clustering and annotation statistics for each sample. Red volumes indicate the total tags which are effective tags for each sample (average: 52342); Blue volumes indicate taxon tags (average： 51338) used to construct OTUs and get annotation information; Green volumes indicate unclassified tags (average: 0) which mean the tags without annotation information; Orange volumes indicate unique tags (average： 1004) whose frequency is 1, and cannot be clustered into OTUs; purple volumes indicate OTUs (average: 262) obtained per sample. The numbers above each volume indicate the tags for each sample.

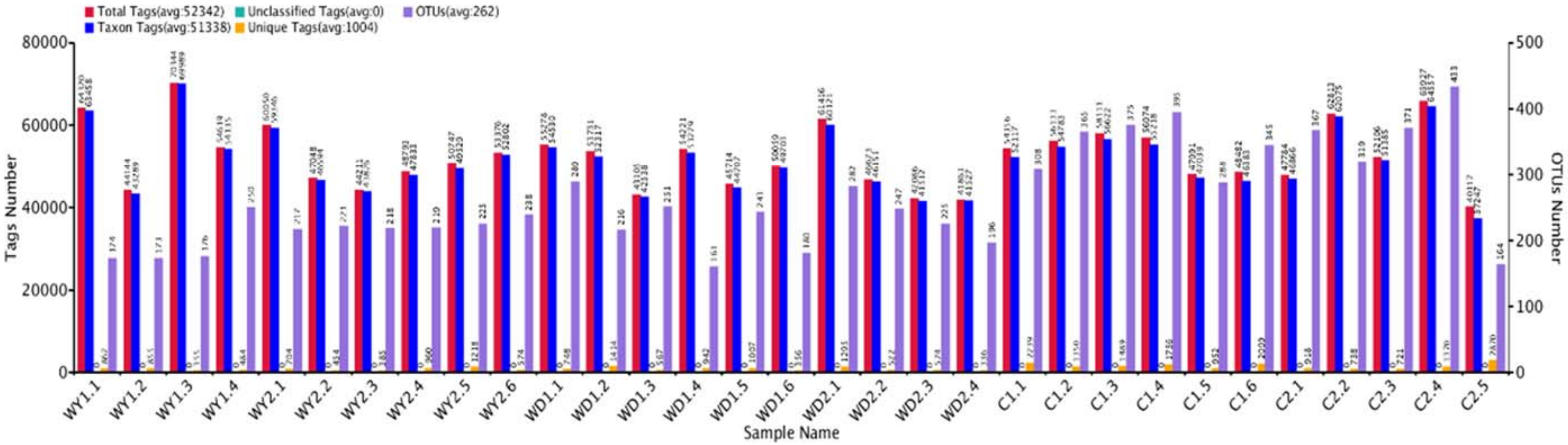

Fig. S2 Rarefaction curves of each sample. Different colors of the lines indicate different sample, respectively.

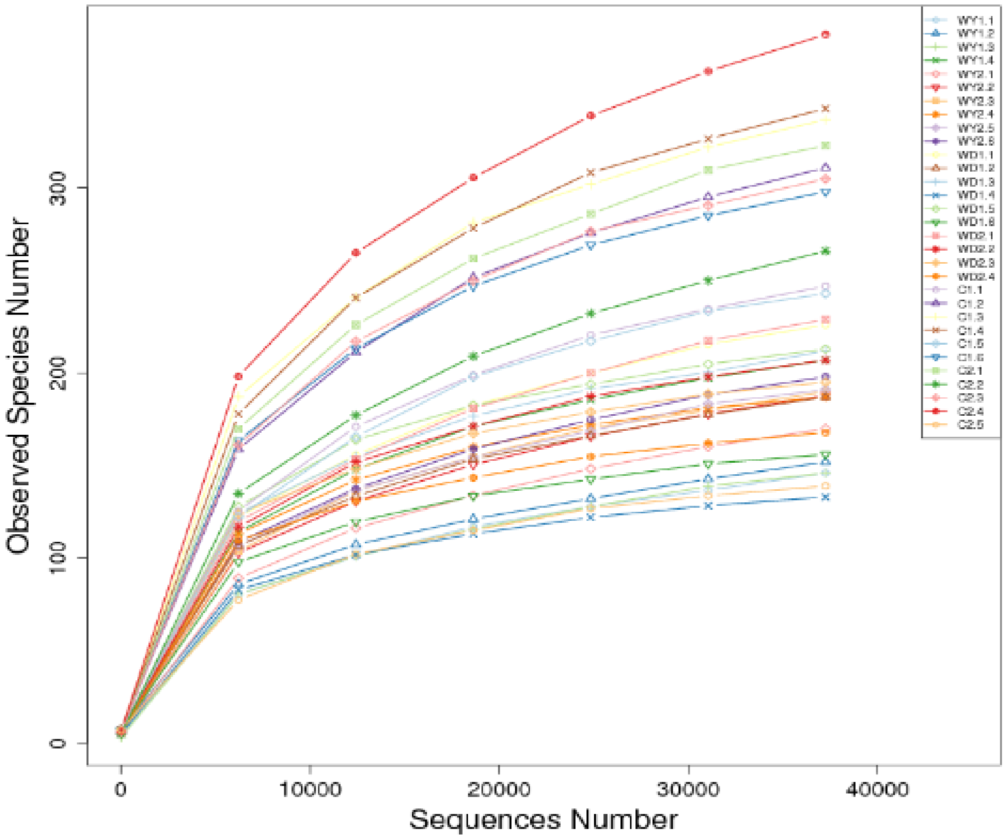

Fig. S3 Species accumulation of samples. Data are expressed as mean ± SEM.

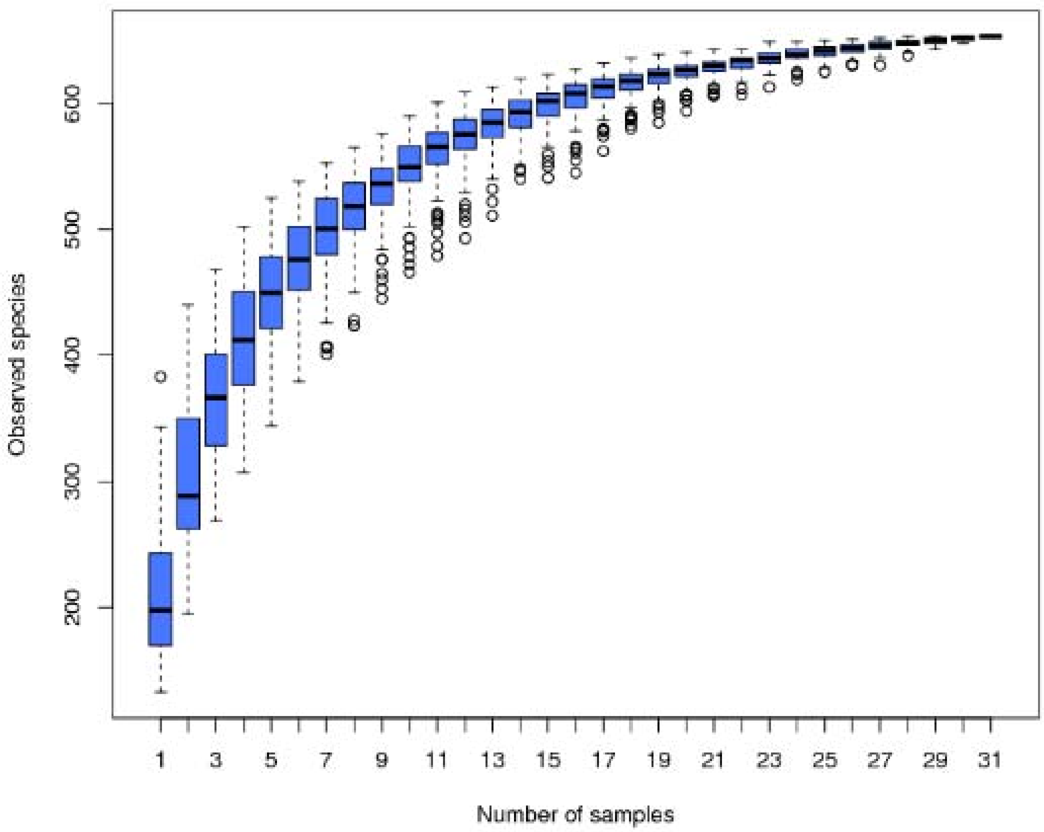

Supplement: Supplementary file 2 [file Data_Sheet_2.pdf]
